# Editorial: From Pathogenic Infections to Inflammation and Disease - the Tumultuous Road of the ‘Cytokine Storm’

**DOI:** 10.3389/fcimb.2021.827151

**Published:** 2022-01-10

**Authors:** Henry Puerta-Guardo

**Affiliations:** ^1^ Collaborative Unit for Entomological Bioassays, Campus of Biological Sciences and Agriculture, Autonomous University of Yucatan, Merida, Mexico; ^2^ Virology Laboratory, Center for Research “Dr. Hideyo Noguchi”, Autonomous University of Yucatan, Merida, Mexico

**Keywords:** cytokine storms, COVID-19, SARS-CoV-2, human metapneumovirus, group A streptococcus, vaccine, antivirals

In the last decades, the incidence of emerging and reemerging infectious diseases have dramatically increased threatening the public health systems worldwide. The appearance of several new pathogens causing disease of marked severity, such as the human immunodeficiency virus (HIV) and other retroviruses, arenaviruses, hantaviruses, Ebola virus and coronaviruses, as well as the simultaneous re-appearance of old pathogens including those that cause cholera, plague, severe dengue, and yellow fever, are having a considerable impact on the global public health and the economy (Anderson and Gray, 2014). Currently, it is estimated that over 25% of human mortality worldwide is attributable to infectious diseases, which translates in 15 million deaths per year mainly occurring among children (<5 years of age) from developing countries (Michaud, 2009). Recently, the rapid spread of a novel severe acute respiratory syndrome (SARS)-like coronavirus (SARS-CoV-2) caused over 200 million of cases and almost 5 million of deaths in an unprecedent pandemic with long-term consequences on human health and global behavior (WHO, 2021). Coronaviruses are a diverse group of viruses infecting many different animals, and they can cause mild to severe respiratory infections in humans (Shereen et al., 2020). This new highly transmissible and pathogenic coronavirus SARS-CoV-2 caused an acute respiratory distress syndrome (ARDS), named ‘coronavirus disease 2019’ (COVID-19), mainly characterized by rapid lung deterioration that results in a robust immunological response triggering a phenomenon called *cytokine storm* that according to several reports contributes significantly to the severity of COVID-19, leading to multiple organ failure and millions of deaths (Castelli et al., 2020; Coperchini et al., 2020; Mahmudpour et al., 2020; Ye et al., 2020).

Cytokines are a broad group of soluble proteins secreted during pathogen infection by infected and non-infected immune cells with both pro-inflammatory and anti-inflammatory effector functions that stimulate or inhibit systematic inflammation (Turner et al., 2014). While many cytokines have beneficial effects facilitating pathogen clearance or promoting tissue repair, other cytokines exert serious harm to the host, inflicting tissue damage (Teijaro, 2017). This latter phenomenon known as *cytokine storm* (Cron and Behrens, 2019; Fajgenbaum and June, 2020), and how it contributes to the development of severe infections diseases with life-threatening consequences in human health, became the main focused of this Research topic called “*From Pathogenic Infections to Inflammation and Disease - the Tumultuous Road of the ‘Cytokine Storm*” which aims to generate and disseminate newest scientific insights to improve the understanding of the role of cytokine storms in promoting immunopathology during bacterial and viral infections. Here, we accepted 3 original scientific manuscripts and 2 reviews focused on the emerging advances in infections caused by distinct animal pathogens and how are these related to the phenomenon of cytokine storm, and the identification of targets for novel therapeutic interventions, and vaccine development.

In the first review by Gomes et al., authors proposed the inhibition of CatL to treat the exacerbated inflammatory response that occur in COVID-19 patients by suggesting the use of distinct commercially available drugs with known anti-inflammatory or potential antiviral activity (Gomes et al.). CatL is a lysosomal cysteine protease that participates in numerous cellular processes, such as apoptosis, antigen processing, and extracellular matrix remodeling (Turk et al., 2012). Under pathological conditions, CatL has been shown to be critical in the development of tumor invasion and metastasis, inflammatory diseases, cardiovascular disorders, renal disease, diabetes, bone diseases, viral infection, and other diseases (Turk et al., 2012). As a host protease, CatL is involved in degrading extracellular matrix, playing a critical role in facilitating or clearing pathogen infections (Turk et al., 2012). This dichotomy is exemplified by CatL helping Hendra virus, SARS coronavirus and Japanese Encephalitis virus (JEV) to invade cells or to cause inflammatory responses and viral pathogenesis, but also CatL being essential in protection against pneumonia caused by mycoplasma or Influenza A (Pager and Dutch, 2005; Mori et al., 2007; Xu et al., 2013; Puerta-Guardo et al., 2016; Xu et al., 2016; Glasner et al., 2017; Puerta-Guardo et al., 2019; Pišlar et al., 2020). In the case of SARS-CoV-2, inhibitors of CatL have shown to reduced SARS-CoV-2 entry *in vitro* and *in vivo* (Simmons et al., 2005; Liu et al., 2020; Shang et al., 2020; Zhao et al., 2021; Ma et al., 2021). Based on these findings and given the rapid growth of this COVID-19 pandemic, the lack of effective antiviral therapies, and the continues threatens poses by the potential emerging of new pathogens like SARS, ready-to-use CatL inhibitors should be explored as a treatment option. Here, Gomes et al., make an interesting proposal to use US FDA-approved drugs with known inhibitory activity against CatL as a possibly safer and potentially effective therapy against SARS-CoV-2 infection and the severe inflammatory responses triggered during COVID-19.

On a second review, Sengunpta et al., hypothesized the potential effect of spice-derived bioactive components in the management of COVID-19 (Sengunpta et al.). Spices are natural dietary components that can provide antiviral effects by reducing oxidative stress and balancing pro-inflammatory and/or anti-inflammatory mediators to maintain cellular and immune homeostasis (Kunnumakkara et al., 2018). Here, the authors proposed how plant derived spices including turmeric, cinnamon, seeds, clove and saffron, bay leaves, chili, and black pepper (Sengunpta et al.) may protect against severe manifestations during COVID-19. This review emphasizes how these bioactive components might alleviate the sustained pro-inflammatory condition observed in COVID-19 by inhibiting the activity of pro-inflammatory and vasoactive molecules such as tumor necrosis factor-alpha (TNF-α), interleukins (IL6, IL8), and chemokine (CCL2) (Sprague and Khalil, 2009); how they may prevent the tissue damage induced by oxidative stress and pro-inflammatory mediators and cytokine storm during SARS-CoV-2 infection, and highlights their effects on the antioxidant pathways to restore oxidative homeostasis and protect from aberrant tissue damage mainly mediated by the nuclear factor erythroid 2-related factor 2 (Nrf2), a transcriptional factor regulating antioxidant genes (Tonelli et al., 2018) and the heme oxygenase 1 (Hmox1), a stress-inducible protein induced by various oxidative and inflammatory signals (Naito et al., 2014). The HMOX1 pathway is known to inhibit platelet aggregation and can have anti-thrombotic and anti-inflammatory properties (Batra et al., 2020); all of which are critical physiological conditions observed in COVID-19 patients (Ali and Spinler, 2021). Spices are essential components of the diet that possess anti-inflammatory, antioxidant, and antiviral properties and importantly, are cytoprotective, preventing tissue damage. Thus, diet may play a significant role in providing beneficial host cell factors that contribute to improve the immunity against deadly SARS-CoV-2 pathogenesis. Although not clinically investigated yet, knowing the beneficial properties of spices and their bioactive compounds, the potential applicability of these plant-derived bioactive molecules against COVID-19 and other viral infections (e.g., pan β-coronaviruses) could be a promising strategy for developing antivirals due to the broad spectrum of their biological activities to target specific cellular pathways involved in viral pathogenesis.

On the other hand, Tan et al., evaluated the safety and efficacy of remdesivir, chloroquine, ivermectin, and doxycycline against the murine coronavirus (MCV), as a safe surrogate model for SARS-CoV-2 infections *in vitro* Tan et al. As SARS-CoV-2, the MCV also known as Mouse hepatitis virus (MHV), is a positive-strand RNA virus that belongs to the group 2 coronavirus (CoV) (Ahmed et al., 2020) or β-coronavirus in the *Coronaviridae* family (ICTV, 2020). Remdesivir, a nucleoside analogue that prevent viral RNA synthesis therefore inhibits viral RNA-dependent RNA polymerase (RdRp) has shown potential antiviral activity against SARS-CoV-2 *in vitro* and on clinical trials against COVID-19 (Agostini et al., 2018; Beigel et al., 2020; Bakowski et al., 2021). In this study, the remdesivir monotherapy had the strongest inhibitory activity against MCV infection and viral RNA replication followed by Ivermectin treatment. In combination with remdesivir, the anti-parasitic drug ivermectin exhibited highly potent synergism to reduce the infection of macrophages followed by the combination with doxycycline, a member of the tetracycline class of antibiotics (Holmes and Charles, 2009). These combinations also reduced the cytokine levels for IL-6, TNF-α, and leukemia inhibitory factor, some of the pro-inflammatory and vasoactive molecules described to be altered in severely hospitalized COVID-19 patients (Kimmig et al., 2020; Mahmudpour et al., 2020), and a group of cytokines related to the phenomenon of *cytokine storm* (Tisoncik et al., 2012). So far, there is no effective antiviral therapy to treat for SARS-CoV-2 infection and COVID-19. Although along the whole COVID-19 pandemic numerous reports have been published describing the potential use of attractive drugs such as ivermectin, doxycycline, hydroxychloroquine, azithromycin, and remdesivir (Yates et al., 2020; Mcfee, 2020; Bhowmick et al., 2021), yet there is controversial of using any of these attractive drug candidates either as monotherapy or in combination. This work by Tan et al. penlights their potential use for the treatment of COVID-19 giving new insights into the generation of more effective drug combinations or new formulations against viral infections.

Another emerging respiratory virus described in this Research topic is the human metapneumovirus (hMPV), a first human member of the *Metaphenumovirus* genus belonging to the *Paramyxoviridae* family (Van Den Hoogen et al., 2001), and a well-known cause of upper and lower respiratory tract infections, particularly in children (Heikkinen et al., 2008). In adults, hMPV is associated with severe infection in patients with pulmonary disease and chronic obstructive pulmonary disease in which it induces a pro-inflammatory immune response that results in no adequate clearance of this pathogen (Guerrero-Plata et al., 2005; Malmo et al., 2016). In this second original manuscript, Soto et al., described how a single dose of the recombinant *Mycobacterium bovis* Bacillus Calmette-Guérin (BCG), co-expressed with the phosphoprotein (P) of hMPV (rBCG-P), induced a protective immune response in mice against hMPV disease (Soto et al.). BCG is a widely used vaccine for tuberculosis (TB) disease in children mainly living in developing countries or endemic areas (CDC, 2016), and the hMVP-P protein is an essential viral cofactor for hMPV replication and cell-to-cell spread (El Najjar et al., 2016). Here, Soto et al., discovered that vaccination of mice with rBCG-P reduced the amount of infiltrated immune cells into the lungs (e.g. interstitial and alveolar macrophages), a main cause of inflammation during respiratory diseases leading to chronic obstructive pulmonary disease and long term inflammation after hMPV infection (Haas et al., 2013; Malmo et al., 2016). rBCG-P also stimulated B cells and plasma cells resulting in an early-robust IgA and constant IgG responses that promoted an efficient viral clearance in naïve mice (Soto et al.). Since first described in 2001, the prevalence of hMPV infections, particularly in the pediatric population, has quickly become recognized as an important cause of respiratory tract disease worldwide (Kahn, 2006; Yi et al., 2019; Rodriguez et al., 2021). hMPV is primarily known as an important cause of lower respiratory tract infections in hospitalized children, in which it causes repeated infections throughout their life, indicating transient immunity (Heikkinen et al., 2008). Increasing evidence show that hMPV is also an important cause of respiratory infections in adults, in which it usually causes mild-to-self-limiting infections (Haas et al., 2013), with complicated clinical courses in the frail elderly and the immunocompromised patients. Thus, a vaccine against hMPV is required to prevent severe disease. To date, several promising vaccine candidates have been developed using reverse genetics for viral attenuation including deletion of viral proteins critical for viral replication steps such as virus attachment or RNA replication (Buchholz et al., 2006; Ren et al., 2015), yet, there is currently no hMPV-specific prevention treatment. Here, Soto et al., demonstrated that protection against hMPV disease in mice can be achieved by using a single and low dose of rBCG-P, which modulate the unbalanced immune response elicited upon hMPV infection, and triggers a robust cellular and humoral response that makes it a promising candidate for further and future clinical trials.

In the last review of this Research topic, Wilde et al., gave an updated view of how infection with group A Streptococcus and its virulent factors cause inflammation leading to disease. Also, authors briefly discussed about the recent developments in potential therapeutics based on targeting inflammation and proinflammatory virulence factors of GAS (Wilde et al.). *Streptococcus pyogenes*, also known as “group A streptococcus” (GAS), is a Gram-positive bacterial pathogen of public health significance, infecting 18.1 million people worldwide, mainly children, and immunocompromised and elderly people, resulting in 500,000 deaths each year (Avire et al., 2021). GAS are strict human pathogens that can cause both non-invasive and invasive, benign, and serious diseases including pharyngitis, impetigo, necrotizing fasciitis, and streptococcal toxic shock syndrome (STSS) among others (Walker et al., 2014). Transmission of GAS commonly occurred *via* respiratory droplets, touching skin sores caused by GAS or through contact with contaminated material or equipment (Avire et al., 2021). GAS diseases are highly prevalent in developing countries, particularly in low socioeconomic areas. A critical point in the epidemiology of GAS is that repeated GAS infections may result in post-streptococcal infections sequalae which results in autoimmune diseases, such as glomerulonephritis poststreptococcal, rheumatic fever, and heart disease (Walker et al., 2014). Although GAS is exquisitely sensitive to penicillin, in the last decades an unexplained resurgence of GAS severe infections has been observed (Kaplan, 1991; Cunningham, 2000). The return of these serious infections and sequelae is strong reminder evidence that biomedical science knows relatively little about the diversity in natural populations of GAS as well as the evolutionary forces that drive temporal variation in bacterial disease frequency and severity. GAS poses many virulence determinants that exhibit overlapping and redundancy in the processes of adhesion and colonization, innate immune resistance, and the capacity to facilitate tissue barrier degradation and spread within the human host (Bessen et al., 2015). Some of these GAS virulence factors such as the secreted cysteine protease Streptococcal pyrogenic exotoxin B (SpeB), the superantigens that act as T cell mitogens, the pore-forming toxins Streptolysin O and S (SLO, -S), and the M protein among others, are well-known to induce an aberrant immune activation leading to inflammation that contribute to disease (Wilde et al.). Although, treatment of GAS infections with antibiotics (e.g., penicillin, clindamycin, etc.) remains useful, GAS antibiotic-resistant strains are continuously emerging, and treatment of toxic and severe invasive disease with antibiotics is not always effective, with mortality rates higher than 50% (Davies et al., 1996). Additionally, no vaccine for GAS is available yet. Here, Wilde et al., briefly discussed the potential use of the intravenous immunoglobulin (IVIG) (Stegmayr et al., 1992; Basma et al., 1998; Kaul et al., 1999), FDA-approved drugs (e.g. nelfinavir) designed for other diseases such as HIV (Maxson et al., 2015), and blockers for cellular specific signaling pathways (e.g. nociceptor neurons) (Pinho-Ribeiro et al., 2018), as adjuvant therapies that may reduce the morbidity associated to GAS *via* promoting beneficial immune responses during STSS. Although more evidence and clinical studies are needed, targeting GAS virulent factors as well as antagonizing the host immune response triggered during severe GAS infections, could be a strategy to enhance host defenses and treat not only GAS but other invasive bacterial infections that triggers a cytokine storm.

## Concluding Remarks

Cytokines are an essential component of the inflammatory response that trigger signaling pathways aiming to mitigate the harmful stimuli caused by a variety of factors including pathogens, damaged cells, and toxic compounds. Cytokines are small proteins secreted by immune cells with both pro-inflammatory and anti-inflammatory activity. Although secretion of cytokines is meant to promote tissue homeostasis/restoration and resolution of the acute inflammation, uncontrolled and aberrant stimuli caused by systemic inflammation sometimes results in hyperinflammatory and pathology events known as *cytokine storm*. Pathogens causing systemic inflammatory response syndrome associated to cytokine storms include many bacterial infections, such as those caused by the group A streptococci as described here by described by Wilde et al., or viral infections including those cause by the new coronaviruses (SARS-CoV-2), and metapneumoviruses described here by Gomes et al., Sengunpta et al., Tan et al., and Soto et al. In these pathogen infections, the acute inflammatory response is significantly marked by the presence of elevated levels of cytokines and chemokines and the recruitment of inflammatory cells that trigger an inflammatory cascade accompanied by abundant tissue damage. This cascade of events has been repeatedly associated with poor prognosis of clinical outcomes and pathogenesis in humans and animal models, mainly related to overwhelming systemic inflammation, hemodynamic instability, and multiple organ dysfunction, sometimes resulting in death. To date, despite all great scientific efforts and promising findings, these emerging infectious diseases continue overwhelming the public health system worldwide as no effective therapy or vaccine yet exist against many of them. In this Research Topic, authors expand our knowledge regarding recent advances in understanding the pathogenesis and potential therapeutic or prophylactic treatment leading the effective management of clinical important human respiratory viruses such as the coronaviruses SARS-CoV-2, the human metapneumovirus, and the group A streptococcus (GAS) in which the phenomenon of cytokine storm could take place leading to life-threatening manifestations. Although understanding of the mechanisms, context, and role of the cytokine storm during immune responses and pathology is constantly evolving, we believe that the manuscripts contained in this Research Topic bring important new data as well as thoughtful insights about how either host-directed or pathogen-directed strategies can be developed and employed in the prevention, diagnosis, and treatment of these emerging infectious diseases. A better understanding of the immunopathology events occurring during the infection with these and other emerging pathogens, increases the chances to propose and to find potent antiviral drugs with improved efficacy in preventing severe viral diseases. It is hoped that the information shared can help guide future research studies in understanding the role of cytokine storm in infectious diseases.

## Author Contributions

The author confirms being the sole contributor of this work and has approved it for publication.

## Conflict of Interest

The author declares that the research was conducted in the absence of any commercial or financial relationships that could be construed as a potential conflict of interest.

## Publisher’s Note

All claims expressed in this article are solely those of the authors and do not necessarily represent those of their affiliated organizations, or those of the publisher, the editors and the reviewers. Any product that may be evaluated in this article, or claim that may be made by its manufacturer, is not guaranteed or endorsed by the publisher.
